# Physiological responses and variation in secondary metabolite content among Thai holy basil cultivars (*Ocimum tenuiflorum* L.) grown under controlled environmental conditions in a plant factory

**DOI:** 10.3389/fpls.2022.1008917

**Published:** 2022-10-21

**Authors:** Panita Chutimanukul, Hathairut Jindamol, Akira Thongtip, Siripar Korinsak, Kanokwan Romyanon, Theerayut Toojinda, Clive Terence Darwell, Praderm Wanichananan, Atikorn Panya, Wilailak Kaewsri, Anyamanee Auvuchanon, Kriengkrai Mosaleeyanon, Preuk Chutimanukul

**Affiliations:** ^1^ National Center for Genetic Engineering and Biotechnology (BIOTEC), National Science and Technology Development Agency, Klong Luang, Thailand; ^2^ Food Biotechnology Research Team, Functional Ingredients and Food Innovation Research Group, National Center for Genetic Engineering and Biotechnology (BIOTEC), Thailand Science Park, Pathum Thani, Thailand; ^3^ Department of Horticulture, Faculty of Agriculture at Kamphaeng Saen, Kasetsart University, Kamphaeng Saen Campus, sNakhon Pathom, Thailand; ^4^ Department of Agricultural Technology, Faculty of Science and Technology, Thammasat University, Rangsit Centre, Khlong Nueng, Thailand

**Keywords:** holy basil, antioxidant, secondary metabolite, photosynthesis, controlled environment, plant factory, light-emitting diodes

## Abstract

Holy basil (*Ocimum Tenuiflorum* L.) is a widely used herb containing several bioactive compounds of interest for the food and pharmaceutical industries. Plant factories using artificial lighting (PFAL) is a modern agricultural system that offers opportunity to improve crop production and stabilizes productivity in many herbal plants. However, little is known about the variation among holy basil varieties that can be cultivated and provide reasonable biomass and bioactive compounds in PFAL. We therefore evaluated 10 Thai accessions and two commercial cultivars in a PFAL (with hydroponic cultivation) to categorize cultivar characteristics by investigating physiological responses and secondary metabolite variation at plant flowering stage. Among Thai varieties, net photosynthetic rate (*Pn*) was significantly highest in varieties OC059 and OC081. The greatest growth and biomass measures were observed in OC064. Antioxidant capacity also varied, with the greatest accumulation of total phenolic compounds (TPC), flavonoids, and antioxidant activity by DPPH assay in OC064, and highest terpenoid content in OC194. The accumulation of major compounds confirmed by showing the highest levels of eugenol in OC057, OC063, OC194, and OC195 and methyl eugenol in OC072 and OC081. The highest α-humulene content was found in OC059. PCA based on physiological responses and secondary metabolites indicate that OC064 was clearly distinguished from other cultivars/accessions. These findings demonstrate variation across holy basil accessions for physiologic responses, antioxidant capacity, and secondary compounds in PFAL. These insights lead to identification of suitable varieties which is the most important step of developing an efficient method for producing high quality raw materials of Thai holy basil for supplying the foods and pharmaceutical industries.

## Introduction

Holy basil (*Ocimum tenuiflorum* L., Lamiaceae) is widely known as an aromatic perennial herb originating from India and distributed across other countries in Asia. Based on morphological characterization, holy basil is classified into two principal common types: green (white) and red holy basil. Green holy basil has green leaves and stems, while red or dark green leaves with purple stems have prominently appeared in red holy basil (Malav et al., 2015). Green holy bail is cultivated commercially and used as a raw or dried material to flavor many foods, particularly sweets, beverages and salads, while red holy basil often used in pharmaceutical, and cosmetic industries (Manikandan et al., 2007; Ngamakeue and Chitprasert, 2016). Several studies have demonstrated antioxidant (Hakkim et al., 2007), anti-bacterial (Viyoch et al., 2006), anti-viral, and anti-inflammatory (Paidi et al., 2021) properties of holy basil performed by its phytochemical components in essential oils.

The essential oil in holy basil is synthesized and stored in glandular trichomes which are located on aerial parts on both leaves and stems (Moghadam et al., 2016; Hikmawanti and Nurhidayah, 2019; Rastogi et al., 2020). Most secondary metabolites in this essential oil are classified into groups of phenylpropanoids and terpenoids (Chutimanukul et al., 2022b). Phenylpropanoids extracted from herbal plant such as flavonoids, phenolic compounds, anthocyanin, and other compounds are natural sources of antioxidants (Al-Owaisi et al., 2014). These phytochemicals can scavenge various radicals such as superoxide anion radicals, hydroxyl radicals, and oxygen-derived free radicals (Hakkim et al., 2007). However, secondary metabolite production is influenced by numerous factors, including growing regions (Rajinikanth et al., 2013), environmental conditions (Harakotr et al., 2019; Chutimanukul et al., 2022b), and stress induction (Khakdan et al., 2017). Moreover, variation in secondary metabolites also depends on plant species and cultivars (Moghaddam and Mehdizadeh, 2015). Finally, total phenolic compound biosynthesis and accumulation are strongly affected by genotypes in *O. basilicum* (Ciriello et al., 2021).

Among several phytochemicals, eugenol and methyl eugenol are regularly reported as the prominent volatile organic compounds (VOCs) accumulated in holy basil (Tangpao et al., 2018; Chutimanukul et al., 2022b; Wongpraneekul et al., 2022), and which vary according to cultivar (Kothari et al., 2004; Renu et al., 2014; Piras et al., 2018). Eugenol is well known for its diverse applications such as antiseptic and antibacterial agent, antifungal, anti-inflammatory, antioxidant, antipyretic, cough, phlegm, chest congestion, diarrhea and used in for foods and teas (Gürbüz and Korkmaz, 2022; Sharma et al., 2022). Methyl eugenol is produced from eugenol by methylation in phenylpropanoid biosynthesis pathway and can be found naturally in many in many types of plants (Lewinsohn et al., 2000). It is widely used as a fragrance ingredient in perfume, flavoring agent in beverages, jelly, ice cream and puddings in food and cosmetic industries. Methyl eugenol has been used in in aromatherapy and alternative medicines (Smith et al., 2002; Burdock and Fenaroli, 2005).

Moreover, the composition ratios between eugenol and methyl eugenol show negative correlation among holy basil accessions (Shiwakoti et al., 2017; Wongpraneekul et al., 2022). For instance, local cultivars from Mississippi showed enriched eugenol content, whereas methyl eugenol content is extremely low (Zheljazkov et al., 2008). In contrast, green and red holy basil growing in Thailand produced methyl eugenol as a major chemical compound at significantly higher levels in red holy basil than in green holy basil (Tangpao et al., 2018).

Photosynthetic characteristics such as net rate, stomatal conductance, transpiration rate, water use efficiency, and electron transport rate are physiological traits indicating plant responses to environmental stress such as water, nutrient deficiency and temperature. (Zhao et al., 2005; Kalisz et al., 2016; Kalamartzis et al., 2020). These physiological responses are useful as criteria for cultivar selection. Previous studies have shown that photosynthetic performance not only varies among plant species but also within cultivars (Dou et al., 2019; Ciriello et al., 2021).

Resulting from historic conventional breeding programs, there are numerous holy basil varieties that have been produced by experimental crosses between cultivars. The influence of genetic factors on physiological responses and secondary metabolite accumulation has been reported for several plants (Kwee and Niemeyer, 2011; Rowshan et al., 2012; Çirak et al., 2013), but there are still few studies of holy basil accessions relative to the total number of varieties (Shasany, 2016; Kumar et al., 2018). Plant factory using artificial light (PFAL) refers to modern agricultural system using advanced technologies for plant cultivation in a closed growing system by systematically controlling the cultivation environment (e.g., light, temperature, CO_2_, humidity and nutrient solution) within a regulated indoor space (Luna-Maldonado et al., 2016; Kozai et al., 2019). PFALs multi-shelf vertical stacking and combine with the hydroponics culture technique to increase the planting acreage of medicinal and horticultural plant (Kozai, 2013; Kalantari et al., 2017). This growing system helps to reduce environmental heterogeneity by absolute control of light intensity, temperature, relative humidity, CO_2_, and nutrient solution concentrations. The LED light sources are used to promote biomass productivity and improve the quality of bioactive secondary metabolites in medicinal plants (Zou et al., 2020; Chutimanukul et al., 2022a).

Thus, the purpose of this study is to evaluate the physiological responses of ten holy basil accessions collected from various locations in Thailand compared with available commercial cultivars (red and green holy basil). In this study, holy basil was grown under artificial light with LEDs (light-emitting diodes) in a PFAL. We investigated secondary metabolite production and antioxidant activity during the harvesting period with an overall objective of facilitating the selection of suitable varieties in Thailand under PFAL cultivation for food production and cosmetics industry use.

## Materials and methods

### Plant material and growth condition

Seeds of ten holy basil, *Ocimum tenuiforum* L., accessions including OC057, OC059, OC063, OC064, OC072, OC081, OC113, OC135, OC194, and OC195 were provided from the Tropical Vegetable Research Center (TVRC), Kasetsart University Kamphaeng Saen Campus, Nakhon Pathom, Thailand alongside commercially available green (G) and red (R) holy basil seeds (Chia Tai Co. Ltd., Bangkok, Thailand). Seeds were sown on a germination sponge containing 300 cells/piece (28.5 × 58 × 3 cm) (ESPEC Corp., Japan) under 150 µmol m^-2^ s^-1^ photosynthetic photon flux density (PPFD) of green LEDs for 16 hd^-1^ photoperiods (Thongtip et al., 2022). After 14 days, all seedlings with fully expanded leaves and roots were transferred to a deep-flow techniques (DFT) hydroponic system under a fully controlled environment in a plant factory using artificial lighting (PFAL), featuring mixing of red, green, blue, and white light LED (AGRI-OPTECH Co., Ltd, Taiwan). The EC and pH of the modified Enshi media solution were set at 1.4 mS and 6.5, respectively. After 14 days after transplantation, EC and pH were adjusted to 2.2 ± 0.33 dS m^-1^ a and 6.5 ± 0.06, respectively until harvesting time. Planting density was 22.5 plant m^-1^ on a hydroponics foam sheet. The environmental conditions in the PFAL were 16 h d^-1^ photo-period with 250 µmol m^-2^ s^-1^, 25.36 °C ± 0.47 temperature, 72.72 ± 2.99% relative humidity (RH), 1036.68 ± 31.46 µmol mol^-1^ CO_2_ concentration and 0.41 ± 0.4 m s^-1^ wind speed. For further details of the daily environmental conditions, see **Supplementary File 1**.

### Plant growth and biomass accumulation

Growth responses and plant biomass were evaluated during the harvesting stage (49 days after transplanting; DAT) with full blooming of flower in whole plant. Growth phenotypes, including plant and canopy height of 12 cultivars/accessions, were measured by Image J software (ImageJ; http://imagej.nih.gov/ij/). Above-ground tissue (stem, leaves, inflorescence) of holy basil were harvested to obtain fresh weight on digital scales. Subsequently, all plants were dried at 40°C for 72 h in a hot air oven and weighed again to ascertain dry mass.

### Photosynthetic capacity and leaf spectral reflectance

Gas exchange parameter responses including net photosynthetic rate (*Pn*), stomatal conductance (*gsw*), transpiration rate (*E*), and intercellular CO_2_ concentration (*Ci*) were measured using a Portable Photosynthesis System (LI-6800 LICOR Inc., Lincoln, NE, USA) on the 12 holy basil accessions at 49 DAT. This device featured a 2 cm^2^ round aperture standard chamber by setting the measured atmosphere at the following conditions: 500 mmol m^-2^ of the molar flow of air per unit leaf area, 25°C of leaf temperature, 70% RH, 1,000 µmol mol^-1^ of CO_2_ concentration. Measurement of light intensity was recorded under 200 µmol m^-2^ s^-1^ leaf surface PPFD on a fully expanded leaf at the third or fourth node from the apex of the plant during harvesting stage. Water use efficiency (*WUE*) was determined by measuring the ratio between *Pn* and *E*. Chlorophyll fluorescence parameters comprising PSII maximum efficiency (F_v_′/F_m_′), photochemical efficiency of PSII (PhiPSII), and electron transport rate (ETR) were also examined. The measurement of gas exchange and chlorophyll fluorescence parameters were measured using samples of the fourth fully expanded leaf from the apex of each plant.

The responses of leaf reflectance spectrum among the 12 cultivars/accessions at 49 DAT were recorded between the ranges of 380 to 790 nm and were calculated at a 1.2 nm resolution by a non-destructive method using PolyPen RP 400 (UV-VIS) (Photon System Instruments, Brno, Czech Republic). The experiment was performed with four biological replicates (four plants of each cultivar per replication), and four holy basil leaves were measured from each plant. Measurements of reflectance spectra were conducted on a fully expanded leaf at the third or fourth nodes from the apex of each individual plant.

### Secondary metabolite quantification

#### Sample extraction

After harvest at 49 DAT, all plant samples were dried in an oven at 40°C for 72 h and subsequently ground with mortar and pestle until forming a fine powder. Plant extraction was then conducted using a modified method (Chutimanukul et al., 2022b) by mixing 10 mg of powder with 5 mL absolute methanol solvent containing 1% HCl. The extracted solution was thoroughly mixed by vortex and incubated at room temperature for 3 h. After incubation, the extracted solution was centrifuged at 12,000 rpm for 5 min using an Eppendorf Centrifuge 5810R with rotor F-34-6-38 (6x125g). The supernatant solution was separated into another microcentrifuge tube (2 mL) and used to verify the content of total phenolics, total flavonoids, and DPPH radical scavenging activity.

#### Total phenolics content

Quantification of TPC in holy basil plants was determined by modified Folin-Ciocalteu colorimetric methods (Chutimanukul et al., 2022b). 200 µl of the extracted solution was mixed with 200 µl of 1 N Folin-Ciocalteu reagent. After 15 min of incubation at 25°C, 600 µl of 7.5% of sodium carbonate (Na_2_CO_3_) was added for neutralization. The absorbance of the solution mix was measured at 730 nm with a spectrophotometer after incubation for 1 h at room temperature (MultiskanSky, Thermo Scientific). TPC was calculated from the standard gallic acid with the range 0-250 µg/mL (250, 125, 62.5, 31.25, 15.63, 7.8, 3.9, 1.9 and 0.97 µg/mL). The gallic acid solution was prepared by dissolving in water to fit the calibration curves to calculate TPC concentration. The result was presented as milligrams of gallic acid equivalent (mg of GAE) per gram DW of the sample.

#### Total flavonoids content

Total flavonoid content was assayed using a colorimetric method followed the procedure described by (Chutimanukul et al., 2022b) with minor modification. The extracted solution with 350 µL was mixed with 75 µL of 5% sodium nitrite (NaNO_2_) in a 1.5 mL microcentrifuge tube and then centrifuged under 25°C at 12,000 rpm for 2 min. After keeping the mixture at room temperature for 5 min, 75 µL of 10% aluminum chloride (AlCl_3_·6H_2_O) was added and thoroughly mixed by a vortex. The mixture was centrifuged as previously and left to stand for 5 min. Finally, 1 M sodium hydroxide (NaOH) was added. The homogenate solution was centrifuged and kept at 25°C for 15 min. A determination of solution absorbance was detected at 515 nm using a spectrophotometer (Multiskan Sky, Thermo, Scientific). Total flavonoid content was calculated based on the standard curve of rutin solution dissolving in dimethyl sulfoxide (DMSO), and concentration was expressed as milligrams of rutin equivalents per gram of DW.

#### Total terpenoids content

Total terpenoid content in holy basil was determined by a modified method (Ghorai et al., 2012); sample extraction was performed by mixing 100 mg fine powder of holy basil in 99.9% methanol. The extracted solution was mixed by vortex and then sonicated for 10 min before incubating in darkness at room temperature for 48 h. The solution was centrifuged at 25°C at 6,500 rpm for 15 min. Then 200 µL of the supernatant solution was transferred to another microcentrifuge tube (2.0 mL) to quantify the total terpenoid concentration by adding 1.5 mL chloroform and mixing the sample thoroughly. After 3 min, 100 µL of concentrated sulfuric acid (H_2_SO_4_) was added without mixing and left at room temperature in the dark for 2 h. A reddish-brown precipitate appeared after incubation and then gently pipetted all reaction mixture supernatant out from each microcentrifuge tube without disturbing the precipitate. 1.5 mL of 99.9% methanol was finally added and vortexed until all precipitate was fully dissolved before centrifuging at 12,000 rpm for 10 min. The absorbance of the solution was determined at 540 nm by spectrophotometer using 99.9% methanol as control. The concentration of total terpenoids was calculated using a regression equation from the linalool standard curve, and the result was shown as milligrams of linalool per gram of DW.

#### DPPH radical scavenging activity

The free radical scavenging activity of holy basil was examined by a minor modified method (Chutimanukul et al., 2022b) using 2,2-diphenyl-1-picrylhydrazyl (DPPH) as a free radical. The extracted solution with 100 µL was pipetted into 900 µL of 0.1 mM DPPH and thoroughly mixed. Then, the mixture was centrifuged at 12,000 rpm for 2 min and kept in darkness at room temperature for 30 min. After incubation, the absorbance of the solution was detected by a spectrophotometer at 515 nm. Trolox was used as a reference antioxidant. The antioxidant activity was shown as the percentage of DPPH scavenging by the equation: ((A_control_-A_515_)/A_control_) x 100 (Yen and Duh, 1994).

#### Anthocyanin content

Anthocyanin content was determined using a spectrophotometer following the method described by Chutimanukul et al., 2022b with minor modification. 50 mg of fine powder was mixed in 600 µL of 1% HCl in methanol and incubated in the darkness at room temperature. After 3 h of incubation, 400 µL of deionized water and 400 µL of chloroform were added and thoroughly mixed before centrifuging at 10,000 rpm for 5 min under 25°C. Then, the supernatant solution of the sample was pipetted to a microplate for detecting the absorbance by spectrophotometer at 530 and 675 nm. The content of anthocyanin was calculated by A_530_-(0.33xA_657_).

#### Volatile compound analysis

To investigate volatile organic compounds (VOCs) content, one hundred milligrams of dried holy basil powder was extracted in 1 mL of methanol solution with 10 μL of gamma-hexalactone (2000 ppm), and vortexed for 1 min in order to extract volatile metabolite compounds. The samples were sonicated for 30 min at 35-40°C using an ultrasonic bath for 30 min at 35-40°C. To remove the insoluble material, the extracts were centrifuged at 10,000 rpm for 5 min at 4°C. The supernatant was collected and stored in a vial at -20°C for further analysis. The prepared solution was used for analyzing the VOCs by gas chromatography coupled with mass spectrometry (GC/Q-TOF).

The analysis of VOCs was conducted using through gas chromatography (Agilent; 7890B) equipped with a quadrupole time-of-flight mass spectrometer (GC/Q-TOF, Agilent, 7250) and PAL autosampler system (CTC Analytics AG, Switzerland). The extract (1 μL) was injected by a multimode inlet (MMI) in a spitless mode, and the inlet temperature was controlled at 250°C. High purity helium (>99.999%) was used as a carrier gas at a constant flow rate of 1.0 mL/min. Gas chromatographic separation was conducted using a DB-FFAP column (30 m x 0.25 mm x 0.25 µm, Agilent Technologies, USA). The GC column temperature program was initiated at 60°C (1 min hold) and then increased to 250°C by 10°C/min (3 min hold). The transfer line temperature was 290°C and total run time was 23 min. The transfer line, ion source (EI), and quadrupole were set as 250°C, 240°C, and 150°C, respectively. Electron ionization (EI) was 70 eV. The mass spectrometer was operated in full scan mode, and the mass range was m/z 20-350 with a data acquisition rate of 5 spectra/s. Quantitative analyses were performed on Agilent MassHunter software (version 10.0 Agilent Technologies, USA), and exported into Microsoft Excel for further data processing.

For each plant cultivar or accession of holy basil, GC/Q-TOF analysis was conducted on the leaves of the whole plant. The target compounds, including eugenol, methyl eugenol, and α-Humulene, were determined. The calibration curves were created with the relative internal response ratio as a function of the analyte concentration of the mixtures of three target compounds ranging from 0.04 to 100 ppm. Retention time and the mass spectra of compound standards were used to confirm the compound identities using commercial reference mass spectra databases (NIST/Wiley version 17 and 11). Qualitative and quantitative analyses were performed on Agilent MassHunter Quantitative Analysis for TOF software (version 10, Agilent Technologies, USA), and exported to Microsoft Excel for further data processing. The quantitative results were reported as µg per gram of DW.

### Statistical analysis

The experiment comprised a completely randomized design (CRD) with four replications (three plants per replication for each accession). Statistical analysis of all data was evaluated by one-way analysis of variance (ANOVA) using IBM SPSS (IBM Corporation; Armonk, NY, USA). The difference between means was analyzed by Duncan’s multiple range test (DMRT) at a significance tested level *p* < 0.05. Results are presented as mean ± SE (standard error). To evaluate the relationships between different parameters; four growth parameters, eight parameters of photosynthetic parameters, five secondary metabolite parameters and three VOCs, and 12 cultivars/accessions, a principal component analysis (PCA) and a hierarchical clustering analysis were performed using statistical software (SAS, Cary, NC, USA). Additionally, a visual assessment of the heat map format was performed to facilitate visual inspection of clustering.

## Results

### Plant growth and biomass production

**Figure 1:**
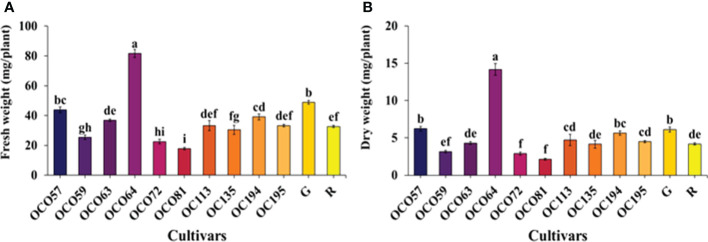
Fresh weight **(A)** and dry weight **(B)** of above-ground tissue of Thai holy basil cultivars/accessions at the flowering stage. R and G represent the red and green commercial cultivars, respectively. Bars represent standard error. Values are represented as mean ± SE (*n* = 4). ANOVA was performed, followed by a mean comparison with DMRT. Letters above bars show the significant difference of means at P < 0.05.

**Figure 2:**
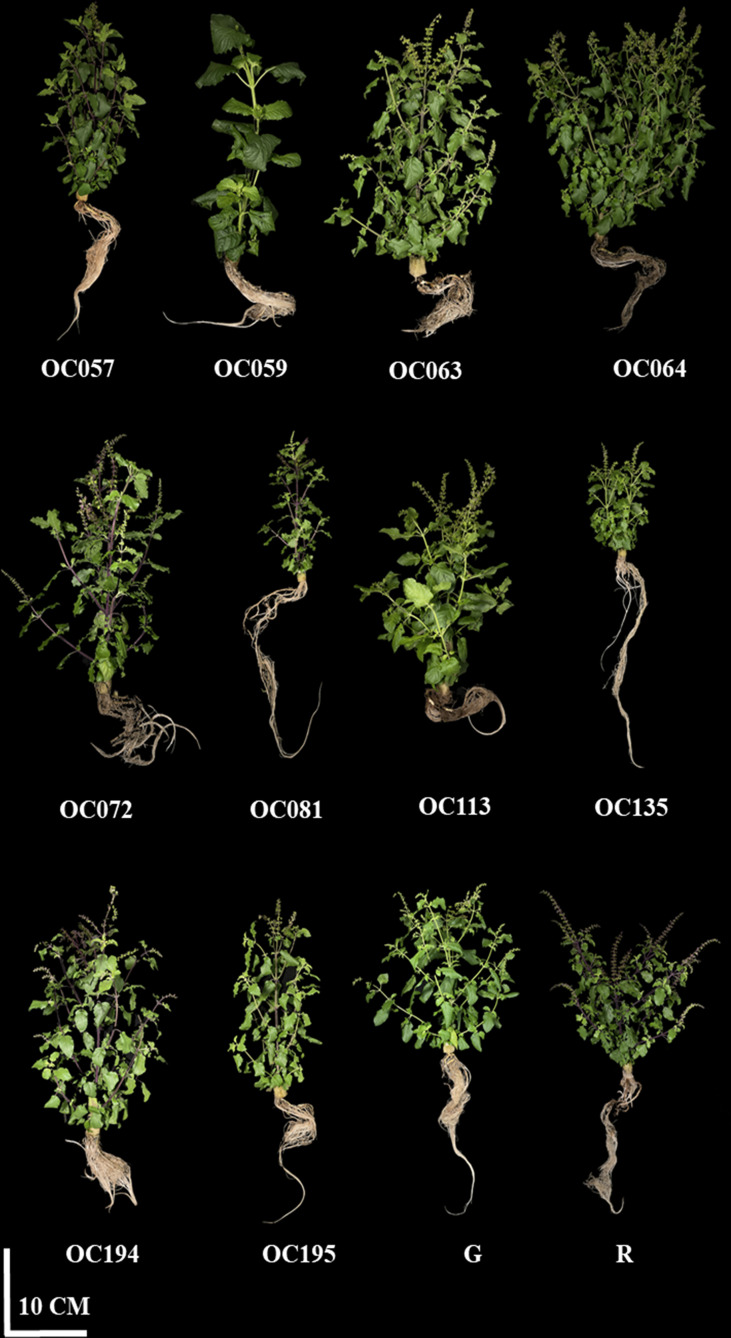
The morphology of Thai holy basil cultivars/accessions at the flowering stage. R and G represent the red and green commercial cultivars, respectively.

Growth characterization of the 12 cultivars/accessions including OC057, OC059, OC063, OC064, OC072, OC081, OC113, OC135, OC194, OC195, alongside the green (G) and red (R) commercial control varieties grown with a hydroponic system under a controlled environment PFAL were determined at the flowering stage (49 days after transplant; DAT) (**Table 1**). Significant differences in growth and morphological characteristics were recorded. OC064 had the highest plant height and plant width. Based on above-ground tissue, shoot fresh weight (FW) was greatest at 81.70 g per plant in OC064 while the lowest value of FW was observed in the commercially available R cultivar. FW of OC064 was 1.64 and 2.507 times greater than R and G commercial cultivars, respectively (**Figure 1**). This was consistent with the results of shoot dry weight (DW), OC064 had the significantly highest DW at 14.17 g per plant, up to 2.31 times greater than the commercial green cultivar and 3.38 times greater than the commercial R (**Figure 1**). Moreover, our study revealed several differences in terms of morphological characteristics among holy basil cultivars/accessions, including stem color, stem pubescence appearance, leaf vein color, sepal color, petal color, and peduncle color (**Supplementary Table 1**). The morphology of all plant cultivars/accessions is displayed in **Figure 2**.

**Table 1:** Growth and plant biomass of 12 cultivars/accessions of Thai holy basil at the flowering stage (49 DAT) in the hydroponic system under a controlled environment PFAL.

Cultivars/Accessions	Plant width (cm)	Height (cm)
OCO57	40.21 ± 0.66b	29.43 ± 0.54cd
OCO59	38.38 ± 1.24bc	19.61 ± 0.37f
OCO63	36.41 ± 0.38cd	30.63 ± 0.79cd
OCO64	42.74 ± 0.59a	37.54 ± 1.27a
OCO72	30.57 ± 0.51e	27.81 ± 1.33cde
OCO81	29.98 ± 0.68e	25.36 ± 0.93e
OCO113	31.56 ± 0.20e	30.47 ± 1.21cd
OCO135	30.12 ± 1.45e	29.10 ± 0.78cd
OCO194	38.67 ± 0.68bc	28.08 ± 0.81cde
OCO195	37.79 ± 0.87cd	27.58 ± 1.03de
G	34.60 ± 0.51d	35.04 ± 0.72ab
R	31.27 ± 0.34e	34.06 ± 0.44b
**F-test**	*	*

Values are represented as mean ± SE (n = 4). Different letters indicate significant differences between cultivations/accessions at p < 0.05*.

### Photosynthetic responses

**Figure 3:**
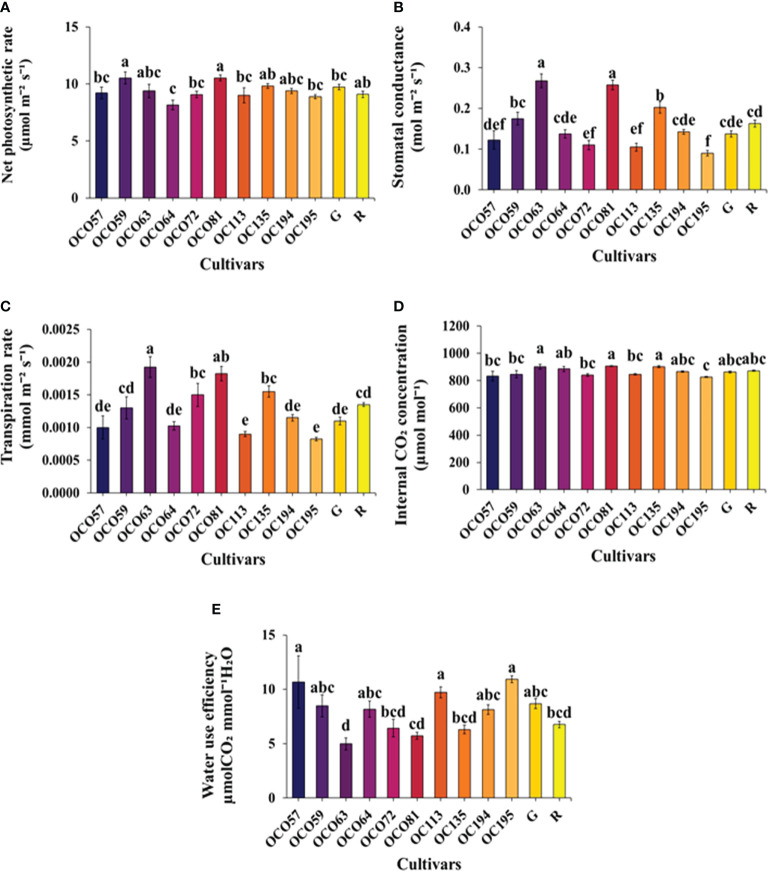
Leaf gas exchange parameters, net photosynthesis rate [*Pn*, **(A)**], stomatal conductance [*gsw*, **(B)**], transpiration rate [*E*, **(C)**], internal CO2 concentration [*Ci*, **(D)**], and water use efficiency [*WUE*, **(E)**] of Thai holy basil cultivars/accessions at the flowering stage. R and G represent the red and green commercial cultivars, respectively. Bars represent standard error. Values are represented as mean ± SE (*n* = 4). ANOVA was performed, followed by a mean comparison with DMRT . Letters above bars show the significant difference of means at P < 0.05.

The study examined the photosynthetic responses of 12 holy basil cultivars/accessions during the flowering stage under a controlled environment in PFAL. The net photosynthesis rate (*Pn*) of all cultivars/accessions was significantly different (**Figure 3A**). Among ten accessions, OC059 and OC081 displayed the highest *Pn*, higher than the two commercial cultivars, while OC064 had a significantly lower Pn value relative to other cultivars/accessions. Furthermore, OC063, OC135, OC194 showed a similar pattern of *Pn* to the green commercial cultivar. During flowering stage, stomatal conductance (*gsw*) showed significant differences among cultivars/accessions (**Figure 3B**). Maximum *gsw* was observed in OC063 and OC081. The lowest reduction in *gsw* value was observed in OC195. Transpiration rate (*E*) among 12 cultivars/accessions was consistent with *gsw* (**Figure 3C**). The Internal CO_2_ concentration (*Ci*) levels of cultivars/accessions differed significantly, with OC063, OC081, and OC135 showing the highest values (**Figure 3D**). Additionally, water use efficiency (*WUE*) of OC057, OC059, OC064, OC113, OC194, OC195 was significantly higher than among four accessions and R cultivar, but it was not significantly different from the green cultivar while the lowest *WUE* value was shown in OC063 (**Figure 3E**).

**Figure 4:**
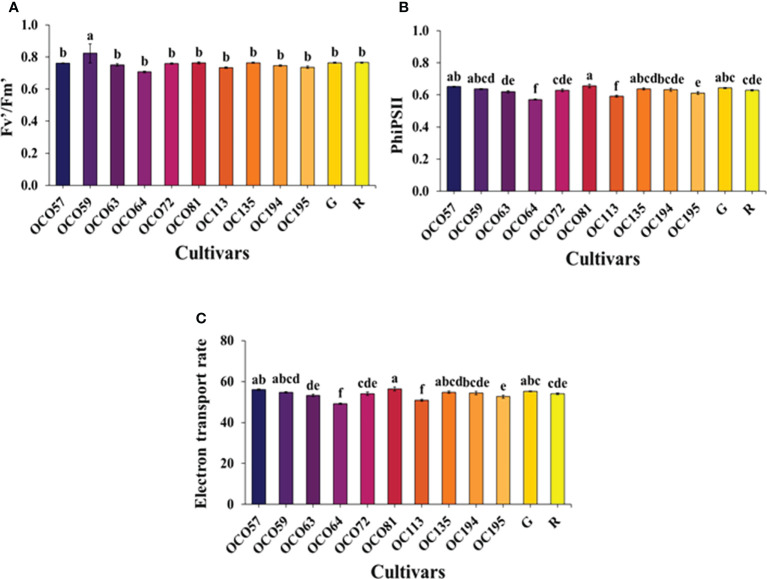
Photosynthetic light reaction parameters, F_v_′/F_m_′ **(A)**, PhiPSII **(B)**, and ETR **(C)** of Thai holy basil cultivars/accessions at the flowering stage. R and G represent the red and green commercial cultivars, respectively. Bars represent standard error. Values are represented as mean ± SE (*n* = 4). ANOVA was performed, followed by a mean comparison with DMRT. Letters above bars show the significant difference of means at P < 0.05.

Light reaction activity, PSII maximum efficiency (F_v_′/F_m_′), photochemical efficiency of PSII (PhiPSII), and electron transport rate (ETR) of holy basil plants at the flowering stage. F_v_′/F_m_′ value were significantly different among 12 cultivars/accessions (**Figure 4A**). A significant difference in PhiPSII was detected (**Figures 4B**). OC081 had the greatest PhiPSII values, while the smallest values were observed in OC064 and OC113. Moreover, OC057 and OC081 recorded a significantly higher level of PhiPSII than the green commercial cultivar. The ETR among 12 cultivars/accessions was consistent with PhiPSII (**Figures 4C**).

During flowering stage, all 12 cultivations/accessions were also investigated at a light saturation point of 1,200 mol m^−2^ s^−1^ of light intensity. Similar responses of photosynthetic parameters and the light reaction activity were found (**Supplementary Figures 1**, **2**).

### Spectral reflectance characteristics

**Figure 5:**
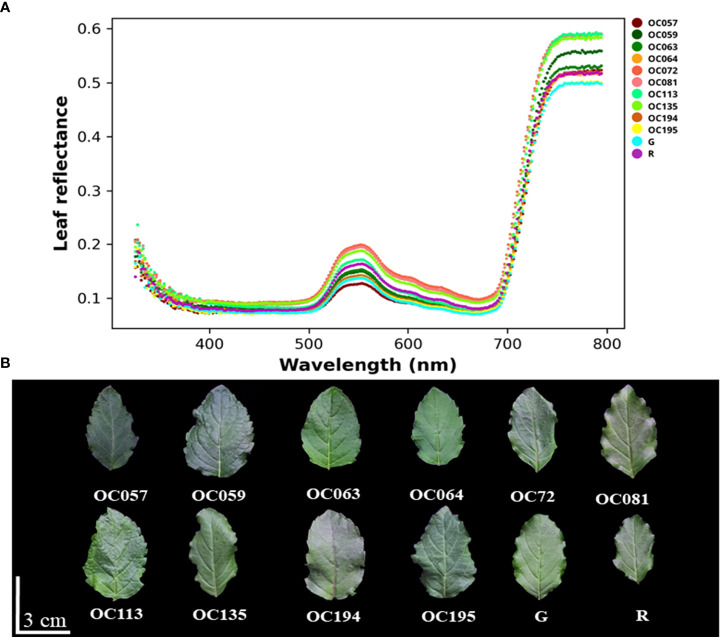
The spectral reflectance **(A)** and leaf characteristics **(B)** among Thai holy basil cultivars/accessions at the flowering stage under a controlled environment in PFAL. R and G represent the red and green commercial cultivars, respectively.

In order to evaluate the leaf reflectance of holy basil plants, the reflectance spectra among Thai cultivars/accessions were compared. The characteristics among Thai holy basil leaves is shown in **Figure 5A**. Leaf characteristics of holy basil used in the spectral data collection are shown in **Figure 5B**. We found that leaf surface spectral reflectance curve (326-794 nm) varied among cultivars/accessions. Overall, all leaf samples revealed a similar spectral pattern through the full wavelength range. Spectral reflectance values of all samples were low, between 390 and 490 nm, which corresponds to the blue spectral range, with 660 and 670 nm in red. In contrast, the spectral reflectance curve slope shows a sharp increase between 700 and 750 nm. Compared to the raw reflectance spectra among these cultivars/accessions, there were two main reflection peaks among leaves, located at 550 nm (green region) and 750 nm (far-red region), respectively. The 530 to 570 nm range featured the high spectral reflection curves of OC72, OC081, and OC135 leaves while OC057 was in the lowest. In addition, a reflection peak in the far-red region (750-794 nm) was observed for OC072, OC081, OC113, and OC135 leaves, whereas the smallest was found in OC195 and the green commercial cultivar.

### Secondary metabolite content and antioxidation capacity

**Figure 6:**
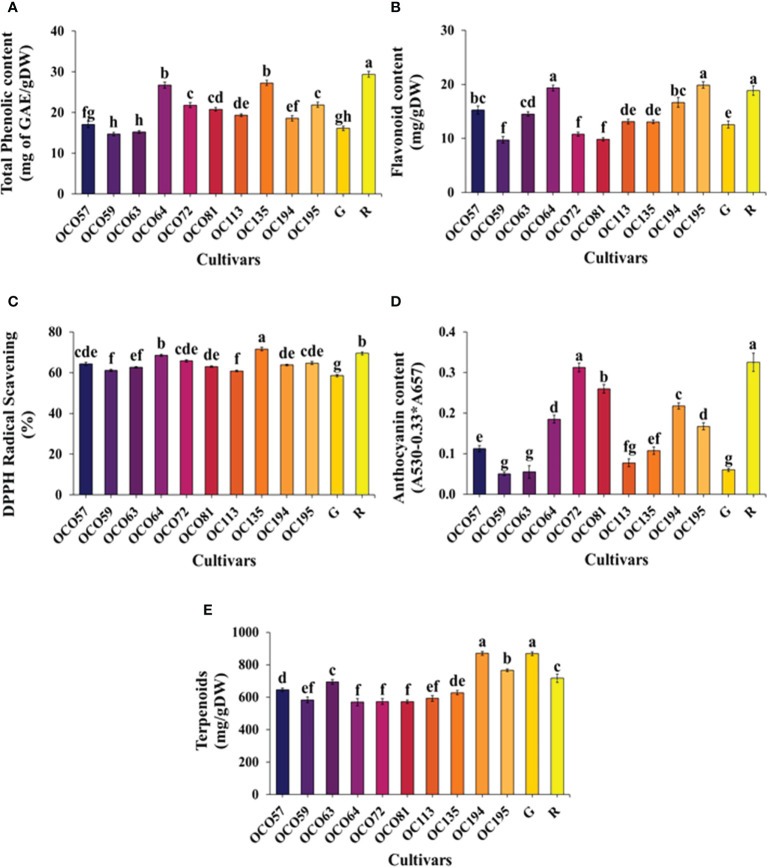
The content of total phenolic compounds (TPC) **(A)**, total flavonoid content (TFC) **(B)**, DPPH **(C)**, anthocyanin **(D)**, and total terpenoids **(E)** of Thai holy basil cultivars/accessions at flowering stage R and G represent the red and green commercial cultivars, respectively. Bars represent standard error. Values are represented as mean ± SE (*n* = 4). ANOVA was performed, followed by mean comparison with DMRT. Letters above bars show a significant difference of means at P < 0.05.

Content of total phenolic compounds (TPC), total terpenoids, total flavonoid content (TFC), DPPH, and anthocyanin of 12 cultivars/accessions at the flowering stage grown under a controlled environment in PFAL are presented in **Figure 6**. Among the ten Thai holy basil accessions, OC064 and OC195 had significantly higher amounts of TPC, while the highest content was recorded in the R cultivar. The lowest levels of TPC were detected in OC059, OC063, and G cultivars (**Figure 6A**). TFC content had similar trends to the TPC result (**Figure 6B**). The highest content of TFC among Thai accessions was highest in OC064 and OC195, followed by OC194, OC057, OC063, OC113, and OC13. A small amount of TFC was found in OC059, OC072, and OC081. The DPPH radical scavenging activities of all holy basil are presented in **Figure 6C**. The greatest DPPH radical scavenging was recorded for OC135, followed by OC064 and the R cultivar. The lowest scavenging activity of DPPH was recorded in the G cultivar. There was significantly different anthocyanin content among cultivars/accessions (**Figure 6D**). Among Thai holy basil accessions, the accumulation of anthocyanin content of OC072 was similar to the R cultivar, displaying significantly higher contents. Further, the lowest accumulation of anthocyanin content was detected in OC59, OC063, and the green cultivar. The highest content of total terpenoids was recorded in OC194 and green cultivar, while the lowest accumulation was in OC064, OC072, and OC081 (**Figure 6E**).

### Volatile compositions

**Figure 7:**
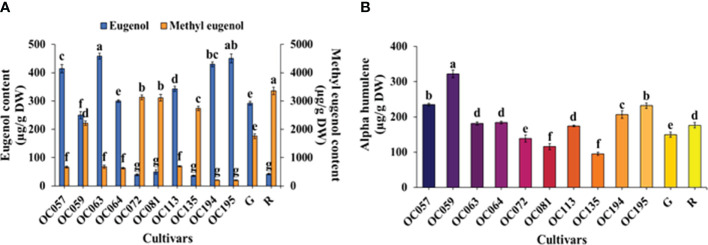
The content of eugenol and methyl eugenol **(A)** and α-humulene **(B)** among Thai holy basil cultivars/accessions at the flowering stage. R and G represent the red and green commercial cultivars, respectively. Values are represented as mean ± SE (*n* = 4). ANOVA was performed, followed by a mean comparison with DMRT. Letters above bars show the significant difference of means at P < 0.05.

Results obtained from GC analyses of three volatile organic compounds (VOCs) are shown in **Figure 7**. Variation among 12 plant cultivars/accessions showed significant effects on eugenol and methyl eugenol in holy basil leaves (**Figure 7A**). Eugenol and methyl eugenol were the major phenylpropanoid group found in all cultivars/accessions. The leaves of OC057, OC063, OC194, and OC195 showed significantly higher eugenol contents than other cultivars/accessions at 4,145.56, 4,581.25, 4,295.41 and 4,506.01 µg g^-1^ DW, respectively. While the level of eugenol was significantly lower in OC072 (392.85 µg g^-1^ DW), OC081 (494.85 µg g^-1^ DW), OC135 (357.54 µg g^-1^ DW), and R cultivar (419.48 µg g^-1^ DW). In contrast, a significantly greater amount of methyl eugenol was recorded in OC072 (3,130.03 µg g^-1^ DW), OC081 (3,115.60 µg g^-1^ DW), and OC135 (2,736.49 µg g^-1^ DW), with the leaves of R cultivar recording the highest methyl eugenol content (3,357.27 µg g^-1^ DW). Moreover, accessions with the smallest amounts of eugenol were OC057, OC063, OC113, OC194, and OC195, ranging from 200.76 to 694.76 µg g^-1^ DW. The amount of α-Humulene found in all the cultivars/accessions ranged from 95.19 to 322.23 µg g^-1^ DW (**Figure 7B**). The α-Humulene as predominant constituents among 12 cultivars/accessions, OC059 had the highest accumulation of α-Humulene content (322.23 µg g^-1^ DW), followed by OC057 (234.91 µg g^-1^ DW) and OC195 (237.8 µg g^-1^ DW), whereas the lowest content of α-Humulene was detected in OC135.

### Principle components and clustering analysis

**Figure 8:**
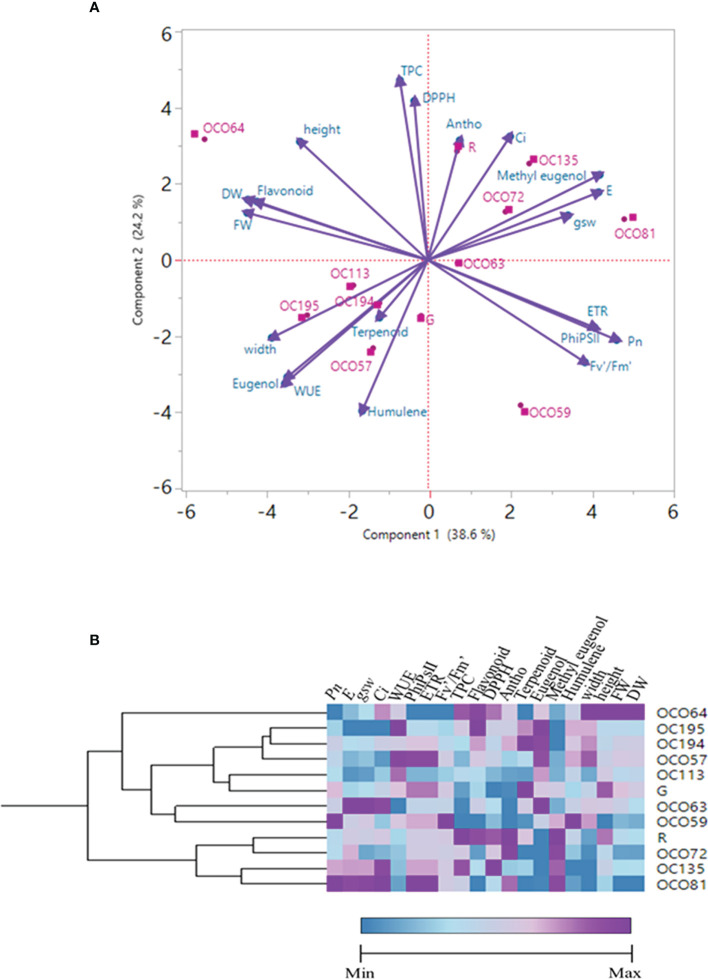
Principal component analysis (PCA) displaying the biplot differentiation **(A)** and Hierarchical clustering analysis of the Euclidean distances **(B)** between four growth parameters, eight parameters of photosynthetic parameters, five secondary metabolite parameters, and three volatile organic compounds of all cultivars/accessions of Thai holy basil.

**Figure 9:**
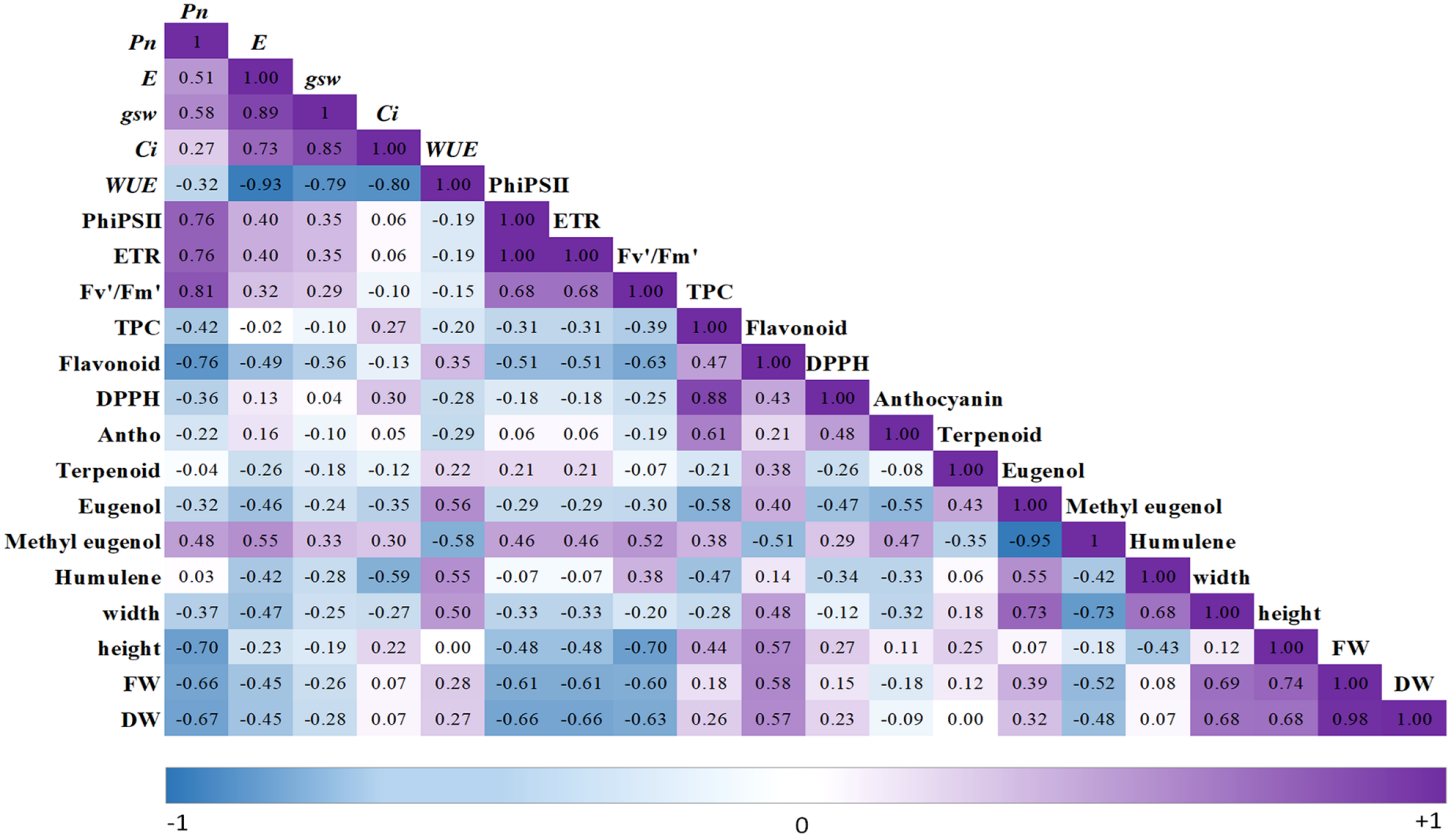
A correlational plot from 20 parameters recorded for 12 holy basils. *Pn*, net photosynthetic rate; *E*, transpiration rate; gsw, stomatal conductance; *Ci*, internal CO2 concentration; *WUE*, and water use efficiency; PhiPSII, photochemical efficiency of PSII. F_v_′/F_m_′, PSII maximum efficiency; ETR, electron transport rate; TPC, total phenolic compounds, flavonoid content; DPPH, radical scavenging, anthocyanin content, total terpenoids content, eugenol content, methyl eugenol content; Humulene, α-humulene content; width, plant width; height, plant height; FW, fresh weight and DW, dry weight.

A principal component analysis (PCA) comparison was performed in order to investigate clustering patterns among 12 cultivars/accessions of Thai holy basil, represented by a biplot including physiological responses and secondary metabolites (**Figure 8A**). Correlations among these traits are shown in **Figure 9**. The two most important principal components (PC) accounted for 62.6% of the variance, PC1 described 37.8%, and PC2 described 24.8% of the total variance. PC1 was positively correlated with Pn, PhiPSII, ETR, and Fv’/Fm’ associated with OC059 while it was negatively correlated with flavonoid content, FW, DW, and height, which were associated with OC064. Furthermore, PC2 was positively correlated with *E*, *gsw*, and *Ci* while it was negatively correlated with *WUE*. Among photosynthetic traits, *E* displayed strong positive correlation with *gsw* (*r* = 0.89, *P* < 0.001), *gsw* positive correlation with *Ci* (*r* = 0.85, *P* < 0.001), and *Pn* positive correlation with Fv’/Fm’ (*r* = -0.81, *P* < 0.001). WUE was also negatively correlated with *E, Ci* and *gsw (r* = -0.93, -0.80, -0.79, respectively, *P* < 0.001). Moreover, among secondary metabolite accumulation, DPPH has a positive correlation with TPC (*r =* 0.88, *P* < 0.001), while methyl eugenol displayed a strong negative correlation with eugenol (*r =* -0.0.95, *P* < 0.001). Hierarchical clustering analysis of the physiological responses and secondary metabolites parameters categorized among Thai holy basil cultivars/accessions is shown in **Figure 8B**. Cultivars/accessions were divided into four main groups. The first cluster showed only OC064, whereas OC195, OC194, OC057, and OC113 were grouped into green cultivars, as shown in the second cluster. Within the third cluster, individuals from accessions from OC059 and OC063 were grouped together. Another main cluster was grouped with R cultivar, OC72, OC135, and OC81.

## Discussion

Holy basil is widely used in Asia as a food product and medicinal herb (Hakkim et al., 2007). Moreover, a constant demand from the food and cosmetic industries has led to plant breeding programs using Thai local accessions. In particular, the cultivation of plants under a controlled environment using a hydroponic system in PFAL increases the further opportunities for enhancement and consistency in quantity and quality in plant production (Kozai et al., 2019). However, the focal plant species is one of the most important factors needing careful consideration under controlled environment cultivation. Growth and secondary metabolite production in holy basil plants were influence under environmental conditions in PFAL (Chutimanukul et al., 2022b). However, the growing of holy basil plant in PFAL was limited by low light intensity rather than light saturation point when compared with outdoor farming (Sourestani, 2016). Thus, the physiological responses, variation in secondary metabolites content, as well as volatile organic compositions (VOCs) are important characters in holy basil selection and improvement for mass production under PFAL. In the present study, we focus on responses of growth and secondary metabolites among various accessions of holy basil under PFAL. Our evaluation among ten Thai accessions and two commercial control cultivars should be of great value for industrialized plant production activities.

Based on plant morphology, holy basil plants differ in plant height, plant width, leaf shape, inflorescence and flower morphology (Agarwal et al., 2013; Malav et al., 2015; Chowdhury et al., 2017; Tangpao et al., 2018). Furthermore, several factors were found to influence the plant growth and responses, as well as the chemical constituents in a plant (Brada et al., 2011; Sims et al., 2014). Several reports have described the important of plants age and harvesting time of holy basil under outdoor farming on large-scale commercial farms by showing the harvesting time during flowering stage (Fuller et al., 2018; Maurya and Sangwan, 2020; Chutimanukul et al., 2022b; Wongpraneekul et al., 2022). Thus, holy basils were examined at flowering stage during the experiment, so as to be relatable to outdoor farm cultivation. This study clearly demonstrates that our panel of 12 cultivations/accessions had several distinct morphological characteristics grown with a hydroponics system under a controlled environment (**Supplementary Table 1**). The highest growth characteristics and plant biomass-related metrics during flowering stage were found in OC064 (**Table 1**), indicating that this accession yields the most desirable growth characteristics relative to other accessions and the two control cultivars (R and G cultivars). In the present study, fresh and dry weight increase was associated with plant height and plant width, a trait phenomenon previously associated with plant growth components (**Table 1**). This was consistent with the reports of (Gutiérrez-Boem and Thomas, 1998), who examined plant growth responses and yields in *Triticum aestivum* L. Moreover, growth parameters often showed strong positive correlations among the 12 cultivars/accessions (**Figure 9**).

Basically, improved growth and biomass among many plants were associated with a higher photosynthetic rate (Kurek et al., 2007; Mateos-Naranjo et al., 2015; Lu et al., 2017; Chutimanukul et al., 2018b; Chutimanukul et al., 2021). The highest *Pn* among Thai local accessions were indicated in OC059 and OC081 (**Figure 2A**). However, both accessions had low values of FW and DW, especially in OC081, which showed the lowest value. In contrast, maximum FW and DW observed in OC064 corresponded with the lowest *Pn* among 12 cultivars/accessions. Our results were consistent with other findings investigating the association between plant biomass and photosynthetic capacity in holy basil (Chutimanukul et al., 2022b) and rapeseed (*Brassica napus* L.) (Shengxin et al., 2016) across various light spectra under PFAL. The negative relationships between photosynthetic parameters (Pn, PhiPSII, ETR, and Fv/Fm) and growth performance (width and height of canopy, FW and DW) were found with correlations (R^2^), being less than 0.7 (**Figure 9**). These suggested that the difference in the productivity of holy basil plants may not be due to the photosynthetic capacity (**Figure 2A**). Stomatal conductance value is one of the key factors influencing photosynthetic rate and affecting changing transpiration rate in plants (Kositsup et al., 2010; Chutimanukul et al., 2018a). Additionally, *WUE* indicates the ratio of water use in plant metabolism relative to water lost through plant transpiration which influences crop yields (Burgess and Huang, 2014; Hatfield and Dold, 2019). In our investigation, we reported the highest *gsw* values among 12 cultivars/accessions, which further positively correlated with higher *Pn*, *E*, and *Ci* and revealed negative correlation with *WUE* (**Figures  3** and **7A**), implying that OC063 and OC081 were able to grow while maintaining photosynthesis response parameters.

Studies in a wide range of plant species have shown photosynthetic light reaction responses that mediate light energy absorption for plant growth (Hichem et al., 2009; Essemine et al., 2011; Shengxin et al., 2016). Photosynthetic light reaction parameters can be used as a performance indicator among photosystems I and II (Murata et al., 2007; Sonoike, 2011; Murchie and Lawson, 2013). In the present study, *Pn* showed a positive correlation with Fv’/Fm’, PhiPSII, and ETR (**Figure 7A**), data that are consistent with prior studies (Shengxin et al., 2016; Chutimanukul et al., 2018b; Chutimanukul et al., 2022b). Additionally, OC081 showed the highest PhiPSII and ETR values among cultivars/accessions, suggesting that this accession may possess excellent adaptation in photosystem II efficiency and ability of the electron transport system under our test conditions, leading to a higher *Pn*.

Variation in reflection spectral characteristics were observed. Moreover, different spectral characteristics of leaf materials is generally maintained in the same plant species at different growth stage conditions (Xie et al., 2013; Mišurec et al., 2016; Zhu et al., 2021). The characteristic responses of the light spectrum reflected in those plants could be estimated by the health status, plant productivity, and plant quality by calculating the ratio of light reflectance characteristics caused by pigment absorption (Gitelson et al., 2005; Buschmann et al., 2012; Kim et al., 2018). In this study, the raw reflectance spectra among leaves showed the same spectral reflectance curves, but the leaf reflectance values were different at each main peak (**Figure 5**). This suggests that light reflectance among cultivars/accessions can also be used to evaluate the possibility of a non-destructive approach for estimating plant status and plant secondary metabolite levels in holy basil.

Holy basil has been used as a medicinal herb for several years in several Asian countries (Dharmani et al., 2004), and previous reports have demonstrated the secondary metabolite compositions in *Ocimum* species under different growth conditions (Joshi, 2013; Patel and Modi, 2018). Phenolic compounds and flavonoids are important plant constituents responsible for scavenging reactive free radicals (Soobrattee et al., 2005; Kumar and Roy, 2018). In the present study, secondary metabolite and antioxidant analysis used 12 cultivars/accessions grown with a hydroponics system under a fully controlled environment in PFAL (**Figure 6**). OC064 recorded the highest accumulation of TPC and flavonoids compared with other Thai local accessions. While the commercial R control cultivar showed the highest in this study. This observation was consistent with the previous study in two holy basil cultivars (Chutimanukul et al., 2022b), in which red holy basil grown in four different light spectra showed a higher level of TPC flavonoids content than green cultivars at flowering stage. Furthermore, OC064 and OC135 show a higher level of DPPH radical scavenging activities when compared with both Thai local accessions and R cultivars, which indicates that OC064 might be an efficient cultivar under a controlled environment. Additionally, anthocyanin is well known as a pigment of plants, and it is classified into flavonoids that contribute to color and antioxidant activity (Gomez-Casati et al., 2013). The highest significant anthocyanin accumulation was found in OC072, showing a similar level to the R cultivar (**Figure 6D**). This result was consistent with clustering analysis suggesting OC072 was classified with the R control cultivar (**Figure 8B**). Interestingly, OC064 and OC195 had higher anthocyanin accumulation than the green cultivar but less than the R control cultivar (**Figure 6D**). However, both OC064 and OC195 show green and red stem colors, while leaf veins are green (**Supplementary Table 1**). Terpenoids are a large and diverse class of plant metabolites with important applications in the food, pharmaceutical, and cosmetic industries (Masyita et al., 2022). During recent decades, numerous studies have reported that terpenoids have a wide range of biological activities and thus have beneficial impact on human health, including antiallergic, antibiotic-resistant bacteria, antifungal properties, anti-inflammatory, anticancer, and antioxidant (Burt, 2004; Pandey et al., 2017; Álvarez-Martínez et al., 2021). This study showed that OC194 featured the highest total terpenoid content around the same level as the green control cultivar when compared with Thai local accessions (**Figure 6E**). Furthermore, OC064 showed maximum plant growth, plant biomass, and antioxidant properties not linked to VOC accumulation.

Several secondary metabolites comprising more than 30 compounds found in essential oil extracted from holy basil belong to phenolic and terpenoids groups synthesized through the phenylpropanoid and mevalonate pathways, respectively (Chutimanukul et al., 2022b; Tangpao et al., 2022). Methyl eugenol and eugenol, target compounds required by several industries, were the main chemical compounds produced such as in holy basil through phenylpropanoid pathway (Vyas et al., 2014). Moreover, flavonoids and anthocyanins are polyphenols are produced by the phenylpropanoid pathway which demonstrates antioxidant biological activities (Carvalho et al., 2013). The primary VOCs in basil plants are phenylpropanoids and terpenoids (Joshi, 2013; Tangpao et al., 2018; Walters et al., 2021). The biosynthetic pathway of phenylpropanoid synthase is negatively correlated with terpene concentration in three cultivars of sweet basil (Iijima et al., 2004). Those VOCs have been reported to have antioxidant and anti-inflammatory agents. Based on our previous research (Chutimanukul et al., 2022b), the major chemical composition present in two cultivars of holy basil consists of phenylpropanoid. The most abundant component in holy basil VOCs is the eugenol and methyl eugenol, ranging around 74-87% under all treatments, while *α*-Humulene was found at low concentrations in holy basil leaves (Miguel, 2010; Jiménez-Estrada et al., 2013; Chutimanukul et al., 2022b). The use of those volatile organic compounds is used in various fields as a functional ingredient of several products such as the food, cosmetic and pharmaceutical industries (Rogerio et al., 2009; El Hadri et al., 2010; Nejad et al., 2017). Our study showed that OC057, OC163, OC194, and OC195 had a greater content of eugenol when compared with other cultivars/accessions while a reduction in methyl eugenol accumulation was found (**Figure 7A**). Interestingly, this finding clearly shows that holy basil leaves from different cultivars/accessions with high eugenol content had a low concentrations of methyl eugenol (**Figure 8A**). This is consistent with the correlation analyses (**Figure 9**) suggesting that these two major compounds displayed a negative correlation. Similar results have been reported in volatile compositions in fresh holy basil leaves (Wongpraneekul et al., 2022). In India, where cultivars of holy basil consist of green and red cultivars, eugenol is the main component of volatile oil (Raina et al., 2013). However, the green and red cultivars of holy basil in Thailand have shown methyl eugenol as the major compound in leaves (Tangpao et al., 2018). In addition, our results suggest that the proportion of major VOCs varied significantly among 12 cultivars/accessions and could not be predicted by the appearance of the leaf or stem color.

Principal component analysis (PCA) was used to visualize the data of overall relationships and correlations between physiological responses and secondary metabolite parameters among the 12 holy basil accessions/cultivars. The PCA revealed that OC064 was clearly split from other cultivars/accessions driven by FW, DW, height, width, TPC, and flavonoids (**Figures 8A, B**). This was consistent with hierarchical clustering of all traits (growth parameters, physiological response, and secondary metabolite content), OC064 was clustered on its own. Analyses for FW and DW among 12 cultivars/accessions of holy basil in PFAL indicated a strong positive correlation with each other at r = 0.98. In contrast, response to *Pn* and FW showed a negative correlation (r = -0.66). Moreover, FW showed a positive correlation with plant height (r = 0.74) (**Figure 8C**). In addition, PCA was performed to separate the physical data from the biochemical data (**Supplementary Figure 3**). Results also showed OC064 clearly had the highest value for plant growth among physical data which consists of plant height, FW and DW of shoot (**Supplementary Figures 3A, B**), while PCA of biochemical data (e.g., TPC, flavonoid and DPPH) showed that OC064 grouped together with OC135 and R, indicating good performance in antioxidant accumulation (**Supplementary Figures 3C, D**). Red holy basil varieties showed higher antioxidant properties and this plant has been used extensively in traditional Asian medicine for treatment of several diseases such as cold, fever, headaches, whooping cough stomach disorders, blood pressure and diarrhea (Prakash and Gupta, 2005; Wangcharoen and Morasuk, 2007; Shasany, 2016). In the present study, the data from correlation analyses between antioxidant properties and volatile organic compounds were not significantly correlated (**Figure 9**). These results suggest that OC064 exhibited high performance in growth traits and antioxidant capacity, but not significant in VOCs content, which might be more a suitable variety for growing and further studies on cultivation technology of holy basil to improve quality of plant production such as VOCs content under controlled environmental conditions in PFAL.

In summary, our investigation of physiological and growth responses and secondary metabolite content among Thai local accessions showed different performances of Thai holy basils in a controlled environment in PFAL which may indicate different utilities to production in food, cosmetics, and pharmaceutical industries. OC0064 showed a higher plant growth potential and antioxidant traits in this study, which resulted in high biomass production. In addition, our findings provide valuable information in the application of PFAL technology. Although some useful basic information for holy basil production in PFAL have been established, further efforts on optimizing other environmental factors of plant growth, light intensity and light spectrum are required to improve the quantity and quality of crop production.

## 5 Conclusion

In the present study, 12 cultivars/accessions of holy basil, including 10 Thai accessions and two commercial control cultivars (green and red cultivars) were evaluated for physiological responses, physiologic responses, antioxidant capacity, and the accumulation of the secondary compounds at flowering stage under fully controlled environment in PFAL. OC059 and OC081 exhibited a similarly high level of photosynthetic responses as red cultivars. Maximum plant growth and biomass production were observed in OC064. The results of the antioxidant properties of holy basil leaf indicated that the total phenolics, flavonoids, DPPH were also highest in OC064. The quantification of volatiles showed the highest accumulation of eugenol in OC063, OC194, and OC195, methyl eugenol in OC072 and OC081, and α-Humulene in OC059. Furthermore, eugenol and methyl eugenol content found among the 12 cultivars/accessions are negatively correlated. This study indicates the importance of diversity among these accessions and provides useful reference resources for secondary metabolite incorporation for the foods and pharmaceuticals industries in PFAL growing of holy basil.

## Data availability statement

The original contributions presented in the study are included in the article/**Supplementary Material**. Further inquiries can be directed to the corresponding author.

## Author contributions

PaC, KM, and TT: Study conception and design. PaC, HJ, and KR, PrC: Data collection. PrC, AT, SK, PW and AA: Analysis and interpretation of results. AP and WK: Performed the GC/Q-TOF analysis. PaC, PrC: Writing original draft preparation. CD: Revised the manuscript. PaC, PrC and CD: Writing review and editing. All authors reviewed the results and approved the final version of the manuscript. All authors contributed to the article and approved the submitted version.

## Funding

This research was supported by the National Center for Genetic Engineering and Biotechnology (BIOTEC), Thailand (P1951788).

## Acknowledgments

The authors would like to thank Miss Jirada Rodsom, Miss Dakanda Mungarsa and Mr. Jakkaphattara Kas-osot for their help in plant factory experiments, and Prof. Dr. Supachitra Chadchawan from Department of botany, Chulalongkorn University for support in Polypen instrument. We are also grateful to Thammasat University Center of Excellence in Agriculture Innovation Centre through Supply Chain and Value Chain and Major of Agricultural Technology, Faculty of Science and Technology Faculty of Science and Technology, Thammasat University for providing technical supports and instrument, and Miss Jirada Rodsom for preparing all figures for this manuscript.

## Conflict of interest

The authors declare that the research was conducted in the absence of any commercial or financial relationships that could be construed as a potential conflict of interest.

## Publisher’s note

All claims expressed in this article are solely those of the authors and do not necessarily represent those of their affiliated organizations, or those of the publisher, the editors and the reviewers. Any product that may be evaluated in this article, or claim that may be made by its manufacturer, is not guaranteed or endorsed by the publisher.
