# Supplementary material for: Physiological responses and variation in secondary metabolite content among Thai holy basil cultivars (Ocimum tenuiflorum L.) grown under controlled environmental conditions in a plant factory
Source: Front Plant Sci. 2022 Oct 21;13:1008917. doi: 10.3389/fpls.2022.1008917 (PMC9634403; doi:10.3389/fpls.2022.1008917)
Supplement: Supplementary file 2 [file DataSheet_2.docx]

**Supplementary Figure**

**
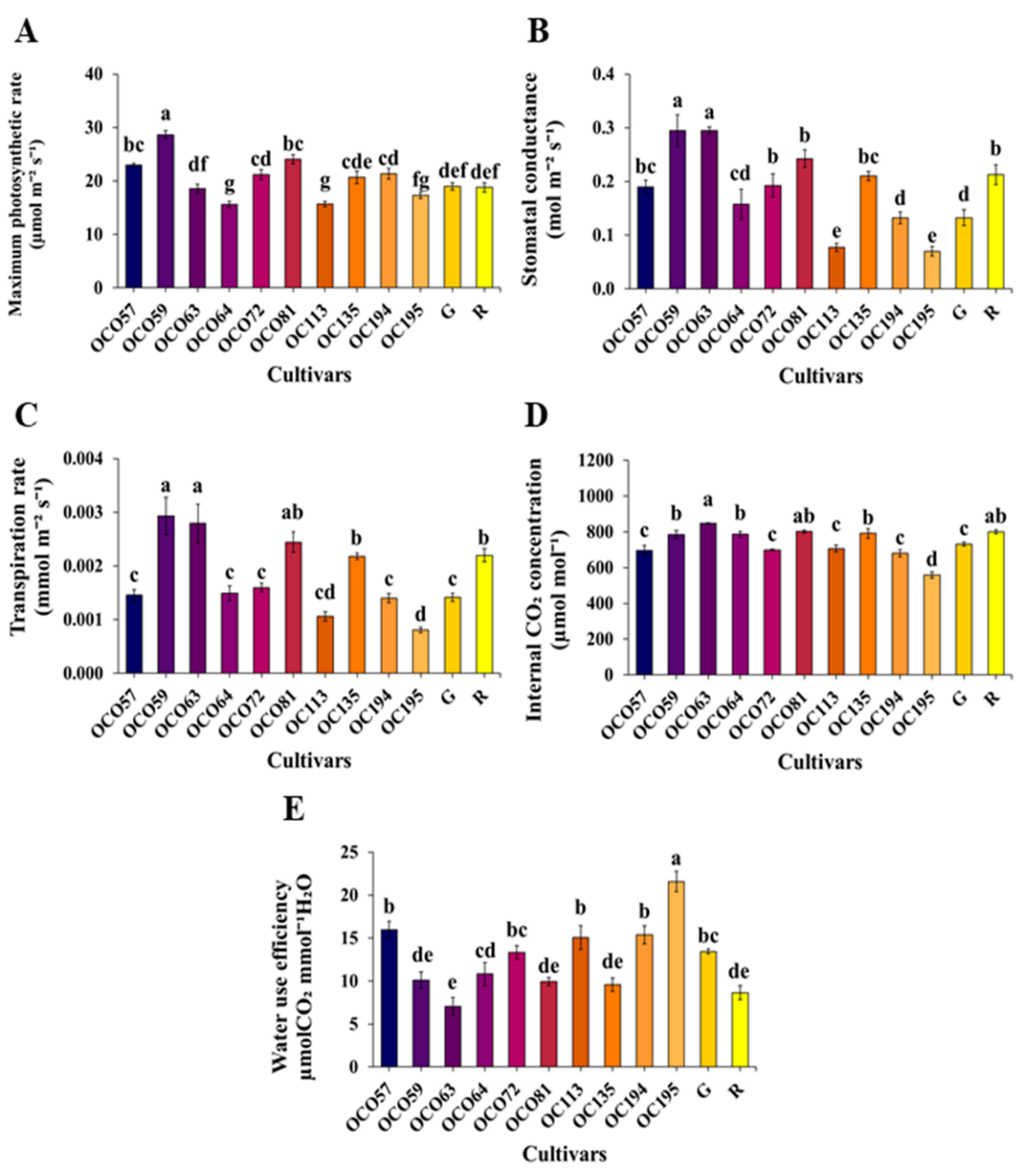
**

**Supplementary Figure 1.** Leaf gas exchange parameters of 12 cultivars/accessions of Thai holy basil using 1,200 mol m^−2^ s^−1^ of light intensity at harvesting stage with hydroponic cultivation under controlled environment in plant factory system. Maximum photosynthesis rate [*A_max_*, (**A**)], stomatal conductance [*gsw*, (**B**)], transpiration rate [***E***, (C)], internal CO_2_ concentration [*Ci*, (**D**)] and water use efficiency [*WUE*, **(E)**]. Bars represent standard error. Values are represented as mean ± SE (*n* = 4). ANOVA was performed followed by mean comparison with DMRT. Letters above bars show the significant difference of means at P < 0.05. “ns” indicates no significant difference.


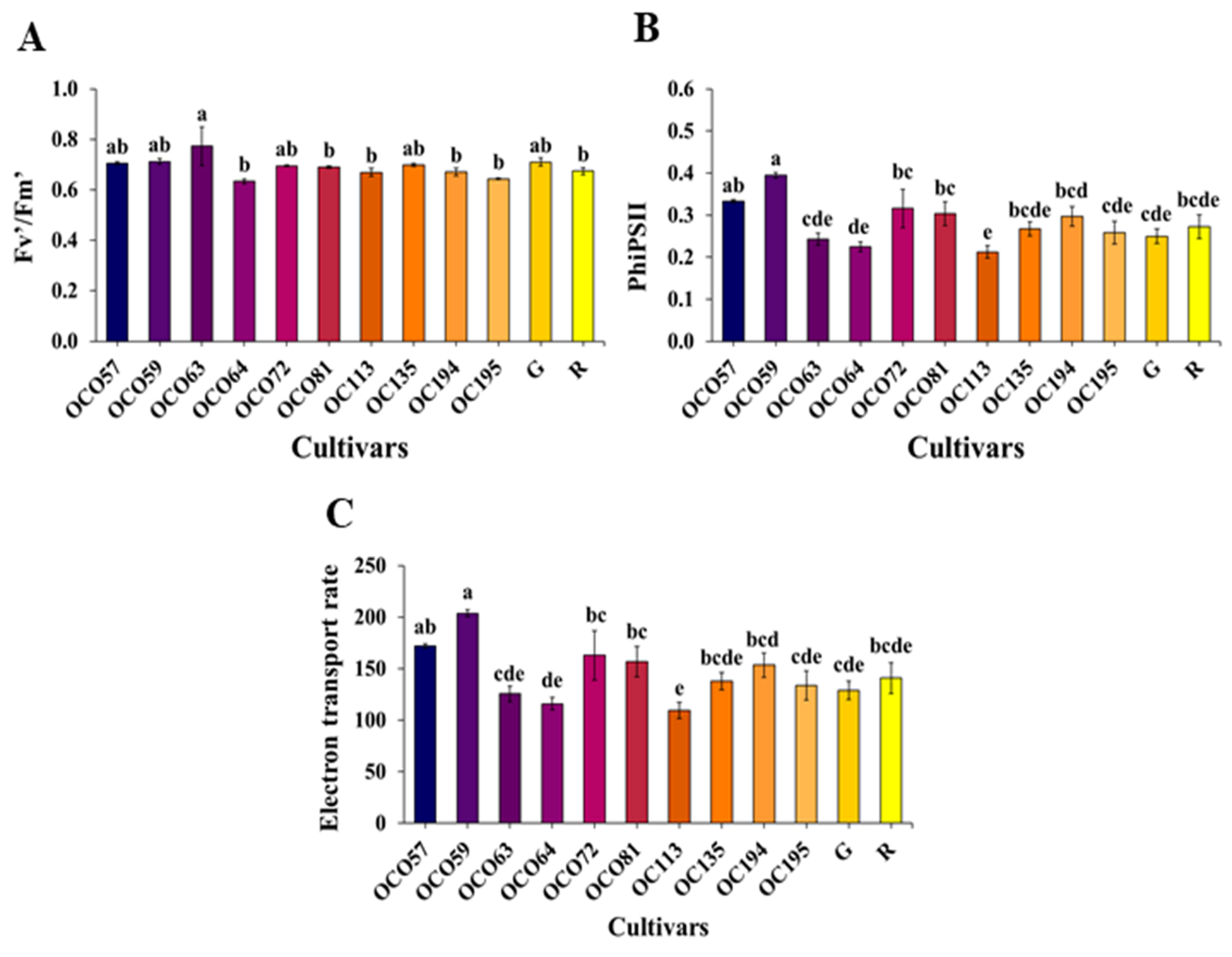


**Supplementary Figure 2.** Photosynthetic light reaction parameters, F_v_′/F_m_′ **(A)**, PhiPII2 **(B)** and ETR **(C)** of 12 cultivars/accessions of Thai holy basil using 1,200 mol m^−2^ s^−1^ of light intensity at harvesting stage at flowering stage was grown with hydroponic cultivation under controlled environment in PFAL. Bars represent standard error. Values are represented as mean ± SE (*n* = 4). ANOVA was performed followed by mean comparison with DMRT. Letters above bars shows the significant difference of means at P < 0.05. “ns” indicates no significant difference.


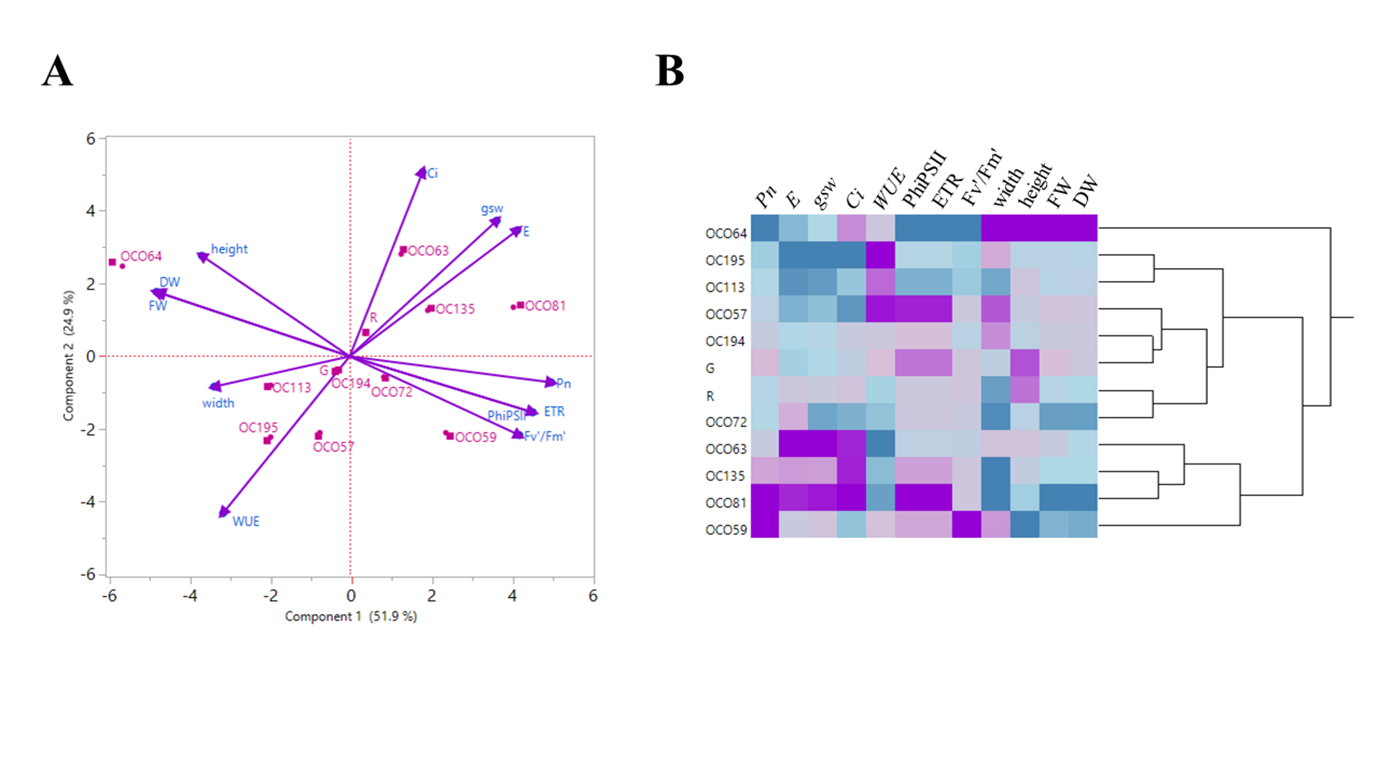

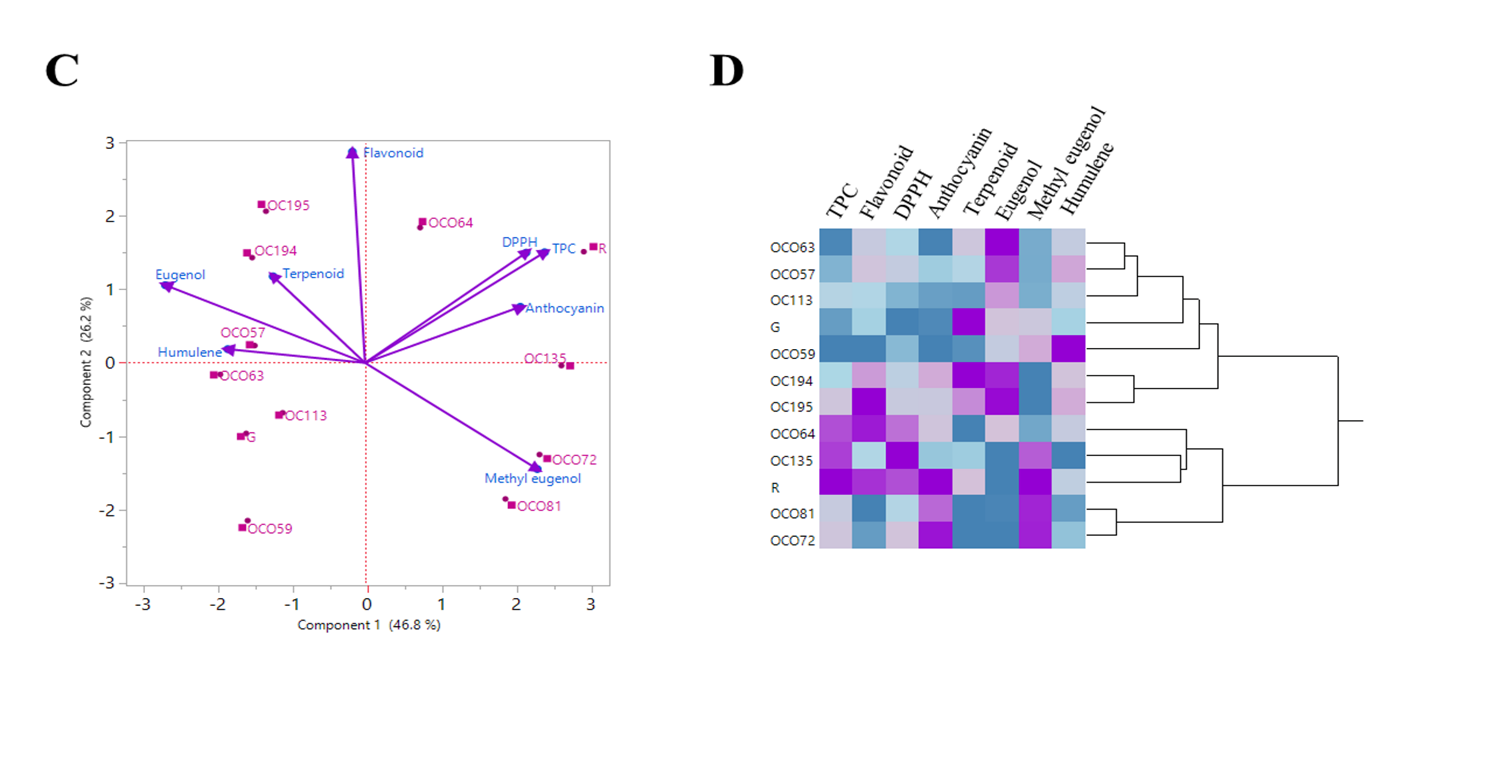


**Supplementary Figure 3.** Principal component analysis (PCA) showing the biplot differentiation **(A)** and Hierarchical clustering analysis of the Euclidian distances **(B)** between four growth parameters (plant width, height, FW; fresh weight, DW; dry weight) and eight physiological data (*Pn*; net photosynthetic rate, *E*; transpiration rate, *gsw*; stomatal conductance, *Ci*; internal CO_2_ concentration, *WUE*; and water use efficiency, PhiPSII; photochemical efficiency of PSII. F_v_′/F_m_′; PSII maximum efficiency, ETR; electron transport rate). The biplot differentiation **(C)** and Hierarchical clustering analysis of the Euclidian distances **(D)** between eight biochemical data (PC; total phenolic compounds, flavonoid content, DPPH; radical scavenging, anthocyanin content, total terpenoids content, eugenol content, methyl eugenol content, Humulene; α-Humulene content) of 12 holy basils.
