# Supplementary material for: Physiological responses and variation in secondary metabolite content among Thai holy basil cultivars (Ocimum tenuiflorum L.) grown under controlled environmental conditions in a plant factory
Source: Front Plant Sci. 2022 Oct 21;13:1008917. doi: 10.3389/fpls.2022.1008917 (PMC9634403; doi:10.3389/fpls.2022.1008917)
Supplement: Supplementary file 3 [file Table_1.docx]

**Supplementary Table 1** Differences in morphological characteristics of 12 holy basil cultivars/accessions at harvesting stage with hydroponic cultivation under controlled environment in plant factory system.

| **Accession**  **no.** | **Plant morphology** | | | | | |
| --- | --- | --- | --- | --- | --- | --- |
|  | **Stem**  **color** | **Stem pubescence appearance** | **Leaf veins color** | **Sepal**  **color** | **Petal**  **color** | **Peduncle**  **color** |
| OC057 | Red | Yes | Red | Green | White | Red |
| OC059 | Green | Yes | Green | Green | White | Green |
| OC063 | Green | Yes | Green | Green | White | Green |
| OC064 | Green + Red | Yes | Green | Green | White | Green |
| OC072 | Red | Yes | Red | Green | White | Red |
| OC081 | Red | Yes | Red | Green | White | Red |
| OC113 | Green | Yes | Green | Green | White | Green |
| OC135 | Green | Yes | Green | Green | White | Green |
| OC194 | Red | Yes | Red | Red | White | Red |
| OC195 | Green + Red | Yes | Green | Red | White | Green |
| Green  Red | Green  Red | Yes  Yes | Green  Red | Green  Green | White  White | Green  White |
